# Alteration of T cell immunity by lentiviral transduction of human monocyte-derived dendritic cells

**DOI:** 10.1186/1742-4690-1-37

**Published:** 2004-11-01

**Authors:** Xiaochuan Chen, Jin He, Lung-Ji Chang

**Affiliations:** 1 Department of Molecular Genetics and Microbiology, Powell Gene Therapy Center, McKnight Brain Institute, University of Florida College of Medicine Gainesville, FL 32610-0266, USA

## Abstract

**Background:**

Dendritic cells (DCs) are professional antigen-presenting cells that play important roles during human immunodeficiency virus type 1 (HIV-1) infection. HIV-1 derived lentiviral vectors (LVs) transduce DCs at high efficiency but their effects on DC functions have not been carefully studied. Modification of DCs using LVs may lead to important applications in transplantation, treatment of cancer, autoimmune and infectious diseases.

**Results:**

Using DCs prepared from multiple blood donors, we report that LV transduction of DCs resulted in altered DC phenotypes and functions. Lentiviral transduction of DCs resulted in down-regulation of cell surface molecules including CD1a, co-stimulatory molecules CD80, CD86, ICAM-1, and DC-SIGN. DCs transduced with LVs displayed a diminished capacity to polarize naive T cells to differentiate into Th1 effectors. This impaired Th1 response could be fully corrected by co-transduction of DCs with LVs encoding interleukin-12 (IL-12), interferon-gamma (IFN-γ), or small interfering RNA (siRNA) targeting IL-10.

**Conclusions:**

DCs transduced with LVs in vitro displayed diminished Th1 functions due to altered DC phenotypes. Our study addresses an important issue concerning lentiviral infection and modification of DC functions, and provides a rational approach using LVs for immunotherapy.

## Background

During HIV-1 infection, an increase in DC-SIGN and CD40 has been reported, as has a decrease in the expression of CD80 and CD86 in dendritic cells (DCs) of lymphoid tissue [1]. Although some suggest that HIV-1 infection reduces the production of IL-12 by DCs,[2] others have shown that DCs derived from HIV-1-infected individuals express both IL-12 and IL-10 at levels similar to those in non-infected individuals[3] While these studies have explored the effects of wild-type HIV-1 on DC functions, the possible effects of HIV-1-derived lentiviral vectors (LVs) on DC functions have not been well characterized [1].

LVs are useful gene transfer tools that can efficiently target many types of cells including DCs. As important immune modulating cells for immunotherapy and vaccine applications, DCs play critical roles in activating the host immune response. DCs can capture, process, and present foreign antigens, migrate to lymphoid-rich tissues, and stimulate antigen-specific immune responses [4]. DCs present a variety of signals to stimulate T cells and initiate immune response; these signals involve multiple signaling mediators, including MHC molecules harboring antigenic peptides (signal 1), the co-stimulatory molecules CD80, CD86, and ICAM-1 (signal 2), and cytokines such as IL-12, IL-4, and IL-10 (signal 3) [5].

Engagement between DCs and T cells not only stimulates T-cell proliferation, but also polarizes differentiation of naïve T helper (Th) cells into IFN-γ-producing Th1 or IL-4-producing Th2 effector cells [6,7]. Production of IL-12 by DCs early in an immune response is critical for polarization of CD4^+ ^T cells toward Th1 function, which is essential for the clearance of intracellular pathogens. IL10, on the other hand, suppresses IL-12 production from DCs and diminishes the commitment of Th1 differentiation. Besides cytokine signaling, there is accumulating evidence that co-stimulatory molecules and adhesion molecules such as CD80, CD86, and ICAM-1 not only engage in T-cell stimulation, but also direct the differentiation of naive T cells [8-10].

Efficient gene transfer into DCs without cytotoxicity has always been difficult [11,12]. LVs transduce DCs at high efficiencies with little to no cytotoxicity, and the transduced DCs retain their immature phenotype, are able to respond to maturation signals, and maintain immunostimulatory potential in both autologous and allogeneic settings [13-16]. In this study, we carefully analyzed cellular response to LV transduction by evaluating changes in DC phenotypes using monocyte-derived DCs prepared from more than 40 blood donors. We investigated the function of DCs to polarize naive T cells to Th effectors after LV infection. Our results demonstrated altered DC functions after LV gene transfer. Most importantly, we illustrated effective modulation of DC immunity by LV expression of different cytokines or siRNA molecules.

## Materials and Methods

### Generation of monocyte-derived dendritic cells

Peripheral blood mononuclear cells (PBMCs) from healthy donors (Civitan Blood Center, Gainesville, FL) were isolated from buffy coats by gradient density centrifugation in Ficoll-Hypaque (Sigma-Aldrich, St. Louis, MO) as previously described [17]. DCs were prepared according to the method of Thurner et al. [18], with the following modifications: On Day 0, five million PBMCs per well were seeded into twelve-well culture plates with serum-free AIM-V medium (Invitrogen Corp. Carlsbad, CA). The PBMCs were incubated at 37°C for 1 hr and the non-adherent cells were gently washed off; the remaining adherent monocytic cells were further cultured in AIM-V medium until Day 1. The culture medium was removed with care not to disturb the loosely adherent cells, and 1 ml per well of new AIM-V medium containing 560 u/ml of recombinant human GM-CSF (Research Diagnostic Inc., Flanders, NJ) and 25 ng/ml of IL-4 (R&D Systems, Minneapolis, MN) was added and the cells were cultured at 37°C and 5% CO_2_. On Day 3, 1 ml of fresh AIM-V medium containing 560 u/ml of GM-CSF and 25 ng/ml of IL-4 was added to the culture. On Day 5, the non-adherent cells were harvested by gentle pipetting. After washing, the DCs were frozen for later use or used immediately.

### Lentiviral vector construction and preparation

MLV and LVs were constructed as described previously [19,20]. The self-inactivating pTYF vectors expressing CD80, CD86, GM-CSF, and IL-12 genes under the EF1α promoter control were constructed by inserting cDNAs that have been previously functionally characterized [21-23]. The cDNA of ICAM-1 was derived from pGEM-T-ICAM-1 kindly provided by Dr. Eric Long. The cDNAs of Flt3L, CD40L, and IL-7 were amplified by RT-PCR using the primers listed below with a modified eukaryotic translation initiation codon (CCACC-AUG): Flt3L sense 5'-TTT CTA GAC CAC CAT GAC AGT GCT GGC GCC AG-3' and antisense 5'-AAG GAT CCT CAG TGC TCC ACA AGC AG-3'; CD40L sense 5'-TTT CTA GAC CAC CAT GAT CGA AAC ATA CAA C-3' and antisense 5'-TTG AAT TCT TAT GTT CAG AGT TTG AGT AAG CC-3'; IL-7 sense 5'AAG CGG CCG CCA CCA TGT TCC ATG TTT CTT-3' and antisense 5'-TTC TCG AGT TAT CAG TGT TCT TTA GTG CCC ATC-3'.

The LVs were produced and concentrated as described previously [20]. Lentiviral siRNA vectors were generated as previously described, using four oligonucleotides. IL-10i#1: sense 5'-GAT CCC CAG CCA TGA GTG AGT TTG ACT TCA AGA GAG TCA AAC TCA CTC ATG GCT TTT TTG GAA A-3' and antisense 5'-AGC TTT TCC AAA AAA GCC ATG AGT GAG TTT GAC TCT CTT GAA GTC AAA CTC ACT CAT GGC TGG G-3'; IL10i#2: sense 5'-GAT CCC CGG GTT ACC TGG GTT GCC AAT TCA AGA GAT TGG CAA CCC AGG TAA CCC TTT TTG GAA A-3' and antisense 5'-AGC TTT TCC AAA AAG GGT TAC CTG GGT TGC CAA TCT CTT GAA TTG GCA ACC CAG GTA ACC CGG G-3' [24].

### Lentiviral transduction of immature DCs and DC maturation

We plated Day-5 immature DCs at 5 × 10^5 ^per well in a 24-well plate containing 200 μl of medium supplemented with GM-CSF (560 u/ml) and IL-4 (25 ng/ml). DC infection was carried out by adding concentrated LVs to the cells at a multiplicity of infection (MOI) of 50–100 (~10^5^–10^6 ^transducing units/ng of p24) as previously described [25]. The infected cells were incubated at 37°C for 2 hr with gentle shaking every 30 min, then 1 ml of DC medium was added and the culture was incubated with the viral vectors for an additional 12 hr. DC maturation was induced by adding lipopolysaccharide (LPS) at a final concentration of 80 ng/ml and TNF-α at a final concentration of 20 u/ml and incubated for 24 hr. To collect mature DCs, the cells were treated with AIM-V medium containing 2 mM EDTA at 37°C for 20 min, and washed three times with PBS.

### Antibody staining and flow cytometry

For analysis of cell-surface marker expression by flow cytometry, we incubated DCs for 10 min with normal mouse serum and then 30 min with fluorochrome-conjugated anti-human monoclonal antibodies. In different experiments, these antibodies included HLA-ABC (Tu149, mouse IgG2a, FITC-labeled, Caltag Laboratories, Burlingame, CA); HLA-DR (TU36, mouse IgG2b, FITC-labeled, Caltag Laboratories); CD1a (HI49, mouse IgG1k, APC-labeled, Becton Dickinson Pharmigen, San Diego, CA); CD80 (L307.4, mouse IgG1k, Cychrome-labeled, Becton Dickinson);CD86 (RMMP-2, Rat IgG2a, FITC-labeled, Caltag Laboratories); ICAM-1 (15.2, FITC-labeled, Calbiochem); DC-SIGN (eB-h209, rat IgG2a, APC-labeled, eBioscience, San Diego, CA); CD11c (Bly-6, mouse IgG1, PE-labeled, BD Pharmigen); CD40 (5C3, mouse IgG1, Cy-chrome-labeled, Becton Dickinson); CD123 (mouse IgG1, PE-labeled, BD Pharmigen); and CD83 (HB15e, mouse IgG1, R-PE-labeled, Becton Dickinson). We included the corresponding isotype control antibody in each staining condition. After two washes, the cells were resuspended and fixed in 1% paraformaldehyde in PBS and analyzed using a FACSCalibur flow cytometer and the CELLQUEST program (Becton Dickinson). The live cells were gated by forward- and side-light scatter characteristics and the percentage of positive cells and the mean fluorescence intensity (MFI) of the population were determined.

### FACS sort of lacZ-positive cells

The lentiviral siRNA vector-transduced cells co-expressing nuclear lacZ gene were separated from un-transduced cells by staining with fluorescent LacZ substrate and sorted by FACS. To label the lacZ-positive cells, we resuspended cells in 100 ul medium and added 100 ul of FDG (fluorescein di-beta-D-galactopyranoside) working solution (2 mM) which was diluted from a 10 × stock FDG solution (20 mM). The stock solution was made by dissolving 5 mg FDG (MW 657, Molecular Probe, Eugene, OR) in a 1:1 mixture of DMSO/ethanol and mixing with ice-cold ddH_2_O to make an 8:1:1 ddH_2_O/DMSO/ethanol solution. The cells were incubated in 37°C water bath for 1–1.5 min, and diluted with 10-fold volume of cold medium and kept on ice until FACS sorting.

### Preparation of naïve CD4+ T cells

The CD4^+ ^T cells from PBMCs were collected by negative selection, using a CD4^+ ^T cell isolation Rosette cocktail (StemCell Technologies, Vancouver, BC) according to the manufacturer's instructions. Briefly, we centrifuged 45 ml of buffy coat (approximately 5 × 10^8 ^PBMCs) in a sterile 200-ml centrifuge tube with 2.25 ml of the CD4^+ ^T cell-enrichment Rosette cocktail at 25°C for 25 min. Thereafter, 45 ml of PBS containing 2% FBS was added to dilute the buffy coat. After gentle mixing, we layered 30 ml of the diluted buffy coat on top of 15 ml of Ficoll Hypaque in a 50-ml centrifuge tube and centrifuged for 25 min at 1,200 *g*. Non-rosetting cells were harvested at the Ficoll interface and washed twice with PBS (2% FBS), counted, and cryopreserved in aliquots in liquid nitrogen for future use. The purity of the isolated CD4^+ ^T cells was consistently above 95%. The CD4^+^CD45RA naïve T cells were purified based on negative selection of CD45RO^- ^cells using the MACS (Miltenyi Biotec, Auburn, CA) magnetic affinity column according to the manufacturer's instructions.

### *In vitro *induction of Th functions and intracellular cytokine staining

The *in vitro *DC:T cell co-culture method was modified based on Caron et al[26]. Briefly, we co-cultured purified naïve CD4 T cells with allogeneic mature DCs at different ratios (20:1 to 10:1) in serum-free AIM-V media. On day 5, 50 u/ml of rhIL-2 was added and the culture was expanded and replenished with fresh AIM-V medium containing rhIL-2 every other day for up to 3 weeks. After day 12, we washed the quiescent Th cells and re-stimulated them with PMA (10 ng/ml or 0.0162 μM) and ionomycin (1 μg/ml, Sigma-Aldrich) for 5 hr, adding Brefeldin A (1.5 μg/ml) during the last 2.5 hr of culture. We then fixed, permeablized, and stained the cells with FITC-labeled anti-IFN-γ and PE-labeled anti-IL-4 mAb (Pharmigen, San Diego, CA). The cells were analyzed in a FACSCalibur flow cytometer (BD Biosciences, San Diego, CA).

### DC-mediated mixed lymphocyte reaction

We co-cultured serial dilutions of DCs, from 10,000 cells per well to 313 cells per well, with 1 × 10^5 ^allogeneic CD4 T cells in a 96-well U-bottomed plate in a total volume of 200 μl for 5 days. The proliferation of T cells was monitored by adding 20 μl of the CellTiter96 solution to each well according to the manufacturer's instruction (Promega). The cells were further cultured for 4 hr before reading the OD_490 _value using a microplate reader (EL808, BIO-TEK Instrument Inc.,Winooski, VT).

## Results

### LVs altered surface marker expression in peripheral blood monocyte-derived DCs

**Figure 1:**
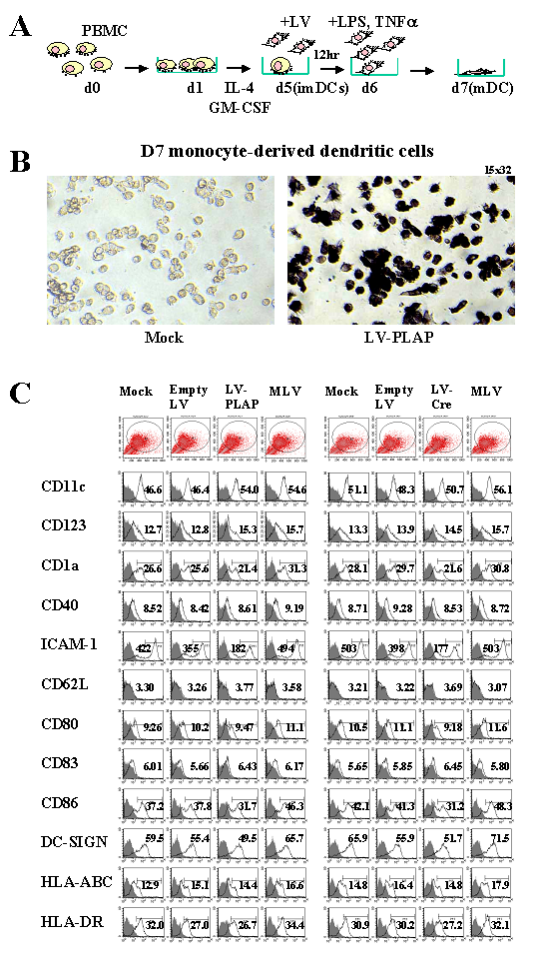
**LV transduction of DCs and analysis of surface marker expression. **PBMC-derived DCs were infected with LV on Day 5 (d5) and after maturation, co-cultured with naïve CD4 T cells for 1–2 weeks before intracellular cytokine staining (ICCS) and flow cytometry (FACS) analysis. The d5 DCs were transduced by LV-PLAP and 48 hr later, analyzed by PLAP enzyme assay. (B) FACS analysis of DC surface markers after viral transduction. The d5 DCs were transduced with LV carrying PLAP or Cre as reporter gene, MLV vectors, empty LV, or no vector controls (mock) and treated with TNF-α plus LPS. The cell surface markers were stained with antibodies and analyzed by FACS. The numbers represent mean fluorescence index (MFI) and the results are representatives of six experiments.

To investigate the effects of lentiviral vectors (LVs) on DCs, we transduced monocyte-derived DCs with LVs encoding different reporter genes. The efficiency of LV transduction of DCs is illustrated by a reporter gene assay (Fig. 1A). The DCs were derived from healthy donors' PBMCs, and on day 5 (d5) of culture, the immature DCs (imDC) were infected with LV-PLAP (encoding placenta alkaline phosphatase). Analysis of PLAP activity on day 7 demonstrated transduction efficiency of > 80% (Fig 1B).

#### DC functions through surface receptor signaling

To see if LVs affected DC surface marker expression, we examined the expression profile of surface molecules on DCs by antibody immunostaining. We transduced PBMC-derived imDC with mock (control 293 supernatants) vectors, empty LV particles, LV, and MLV carrying a reporter gene. After induction of maturation with LPS plus TNF-α, we harvested the DCs for antibody staining and FACS. The results are shown in Fig. 1C and summarized in Table 1. Among the surface molecules tested, CD1a, CD80, CD86, ICAM-1, and DC-SIGN were down-regulated after LV transduction, but not after transduction with empty LV or MLV. The same result was obtained using different preparations of LVs carrying either PLAP or Cre as the reporter gene.

**Table 1:** Surface marker profile of DCs transduced with LVs or MLV vectors.

	Geometrical Mean Fluorescence ± SD			
Surface Marker	Mock	Empty LV	LV	MLV

CD11c	48.8 ± 3.2	47.2 ± 1.3	52.3 ± 2.3	55.3 ± 1.1
CD123	13.0 ± 0.4	13.4 ± 0.8	14.9 ± 0.6	15.7 ± 0.1
CD1a	27.3 ± 1.1	27.6 ± 2.9	21.5 ± 0.2*	31.0 ± 0.3
CD40	8.6 ± 0.1	8.9 ± 0.6	8.6 ± 0.1	9.0 ± 0.3
ICAM-1	462.6 ± 57.5	376.5 ± 30.1	179.5 ± 3.4***	498.5 ± 6.9
CD62L	3.3 ± 0.1	3.2 ± 0.03	3.7 ± 0.1	3.3 ± 0.4
CD80 (B7-1)	9.9 ± 0.9	10.6 ± 0.7	9.3 ± 0.2*	11.3 ± 0.4
CD83	5.8 ± 0.3	5.8 ± 0.1	6.4 ± 0.01	6.0 ± 0.3
CD86 (B7-2)	39.6 ± 3.5	39.6 ± 2.5	31.4 ± 0.4*	47.3 ± 1.5
DC-SIGN	62.7 ± 4.5	55.7 ± 0.4	50.6 ± 1.5*	68.6 ± 4.1
HLA-ABC	13.9 ± 1.3	15.8 ± 1.0	14.6 ± 0.3	17.2 ± 0.9
HLA-DR	31.5 ± 0.8	28.6 ± 2.2	26.9 ± 0.4	33.2 ± 1.7

Results are presented as geometrical mean fluorescence after FACS. Asterisks (*) denote significance of difference by Student t-test (*P < 0.05, **P < 0.01, ***P < 0.001).

### LV transduction impaired DC-mediated Th1 immunity

It has been reported that retroviral infection induces up-regulation of Th2 cytokines including IL-10 and impairs DC maturation [27,28]. Because HIV causes immune suppression and the preceding results showed that LV infection altered the surface marker expression profile of DCs, we suspected that LV infection might also affect DC activation of T cells. To test this, we set up an *in vitro *immunity assay using co-culture of human DCs and naïve T cells.

**Figure 2:**
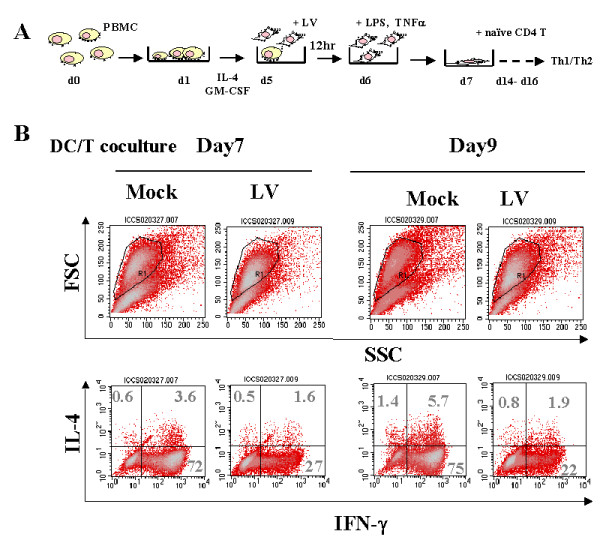
**Impaired Th1 response induced by LV-transduced DCs. **We analyzed T helper function by using DC:T cell co-culture and IL-4 and IFN-γICCS. Immature DCs were infected with mock (293T supernatants) or LV on d5 and treated with LPS and TNFα. The DCs were harvested and co-cultured with naïve CD4+ T cells at a DC:T cell ratio of 1:10. On Day 7 amd 9 after co-culture, the cells were re-stimulated and the T helper cell populations were examined by INF-γ and IL-4 antibody ICCS as described in the Materials and Methods. The percentages of cell populations are indicated in the FACS quadrants. The results are representative of four independent experiments.

We generated DCs from PBMCs and infected the d5 DCs with LV carrying a reporter gene. To characterize the function of DCs, we purified naïve CD4^+ ^T cells from healthy donors' blood and co-cultured the T cells with allogeneic monocyte-derived DCs treated with TNFα and LPS to induce maturation, as illustrated in Fig. 2. The co-cultured T cells were allowed to expand and rest for more than 7 days after DC priming. To analyze Th response, on days 7 and 9 we reactivated the resting T cells with ionomycin and PMA, and subjected the T cells to ICCS using antibodies against IFN-γ and IL-4. We found that the IFN-γ-producing Th1 cell populations were dramatically reduced when incubated with DCs transduced with LVs, from 72% (day 7) and 75% (day 9) for the control to 27% (day 7) and 22% (day 9) for the LV-transduced DCs. The Th2 populations remained essentially unchanged (Fig. 2). In naïve T cells the Th1 response is regulated by the "master transcription regulators" T-bet and GATA-3.[29] Analysis of T-bet and GATA-3 expression in T cells after coculture with LV-transduced DCs showed decreased expression of both T-bet and GATA-3 RNA, and the relative T-bet expression correlated with the Th differentiation according to ICCS of T cells after 8 days of co-culture (data not shown).

### Up-regulation of CD80 and CD86 expression did not restore DC functions

**Figure 3:**
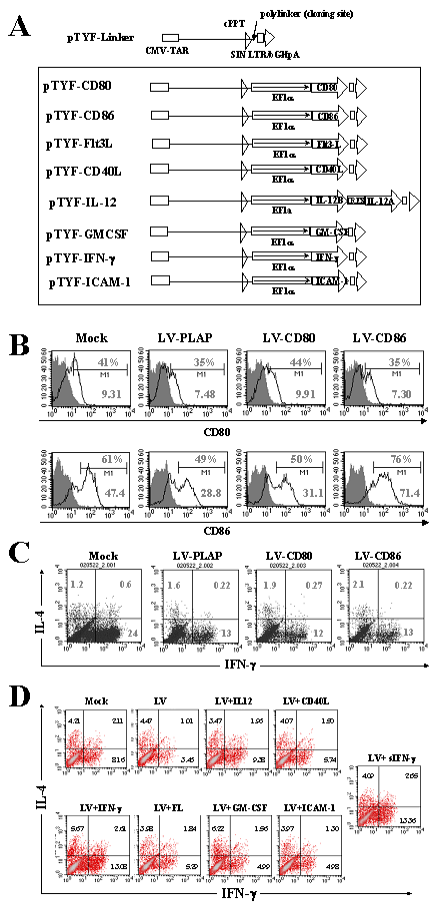
**LV modification of DC immune functions. **(A) Diagram of LV constructs containinging different immune modulatory genes. (B) Up-regulation of T cell costimulators in DCs transduced with LV-CD80 or LV-CD86. Immature DCs were transduced with mock, LV-PLAP, LV-CD80, or LV-CD86 for 12 hr, induced to mature, and analyzed 24 hr later using anti-CD80 and anti-CD86 antibodies. The mean fluorescence intensity and percentage of positive cells are shown. (C) Th1/Th2 assay of DCs with up-regulated CD80 or CD86. The T-cell activation function of DCs was analyzed by DC:T cell co-culture. ICCS and FACS for T helper function were performed 8 days after co-culture. The percentages of different T-cell populations are shown. (D) Th1/Th2 assay of DCs co-transduced with different LV immune modulatory genes. DCs were transduced with LV (LV-PLAP), and co-transduced with LVs encoding different immune modulatory genes, including IL-12, CD40L, IFN-γ, FL, GM-CSF, and ICAM-1, or incubated with soluble IFN-γ. DCs were then treated with TNF-α and LPS and co-cultured with naïve CD4 T cells. The T cells were analyzed for IL-4 and IFN-γ expression by ICCS and FACS 9 days after co-culture. The percentages of different T cell populations are shown in the quadrants. The results are representative of six independent experiments.

Because T cell co-stimulatory molecules are important mediators of DC functions, the down-regulation of CD80 and CD86 in DCs after LV transduction might contribute to the observed Th1 impairment. To examine this possibility, CD80 and CD86 were up-regulated in DCs using LVs encoding these two genes to see if the impaired Th1 response could be corrected. The LVs encoding human CD80 and CD86 were constructed as shown in Fig. 3A. The functions of these CD80 and CD86 genes have been previously demonstrated in an *in vivo *study [21]. DCs were transduced with LVs expressing a reporter, CD80 or CD86 gene, and then treated with LPS and TNF-α 12 hr later. Thirty-six hours after LV transduction, we analyzed the transduced DCs for CD80 and CD86 expression by FACS, using anti-CD80 and anti-CD86 antibodies. The results were consistent with our earlier findings; CD80 expression was reduced from 41% to 35% after LV-PLAP infection, while CD86 expression was reduced from 61% to 49% (Fig. 3B). Their expression was up-regulated after transduction with LVs encoding CD80 and CD86; the expression of CD80 was up-regulated from 35% to 44%, and the expression of CD86 from 49% to 76%.

To see if the up-regulation of the T-cell co-stimulatory molecules in DCs could restore the Th1 response, we co-cultured naïve CD4 T cells with DCs transduced with mock, LV-PLAP, LV-PLAP plus LV-CD80, or LV-PLAP plus LV-CD86. After 8 days, the T cells were reactivated and analyzed by ICCS and FACS using anti-IL-4 and anti-IFN-γ antibodies as described earlier. We found that after LV transduction the Th1 population was reduced from 24% to 13%. Moreover, this impairment could not be corrected by the up-regulation of CD80 and CD86 in DCs (from 13% to 12% and 13%, respectively, Fig. 3C).

### Modification of DC immunity by LVs encoding immune modulatory genes

Cytokine signaling is important in DC-mediated Th differentiation; for examples, IL-12 is critical to Th1 development, and Flt3-ligand (FL) has been shown to enhance IL-12 production in DCs [30]. To overcome the impaired Th1 response after LV transduction, we investigated whether modification of the local cytokine environment in the DC:T cell synapse could promote a Th1 response. LVs expressing different cytokine and receptor genes, including FL, GM-CSF, IL-12 (a bi-cistronic IL-12A and IL-12B construct), CD40L, IFN-γ, and ICAM1 were constructed (Fig. 3A). Expression or function of these different immune modulatory genes has been previously demonstrated. [21-23] DCs were transduced with LVs carrying reporter gene PLAP either alone or co-transduced with different immune modulatory genes. As positive control, we treated DCs with soluble IFN-γ before maturation and DC:T-cell co-culture. The Th function of the LV-transduced DCs was analyzed by DC:T cell co-culture followed by ICCS and FACS analysis of IFN-γ and IL-4, as described earlier. The results showed that LV transduction alone reduced IFN-γ-producing Th1 cell population as found above, from 8.16% to 3.46%. However, co-transduction with LV encoding IL-12 enhanced Th1 response from 3.46% to 9.38%, while co-transduction with LV encoding IFN-γ increased such response from 3.46% to 13.08%, an increase that was similar to that produced by soluble IFN-γ (Fig. 3D). LVs expressing FL, GM-CSF, CD40L, or ICAM-1, on the other hand, exhibited no significant effect.

### Modulation of DC function by LVs expressing siRNA targeting IL-10

**Figure 4:**
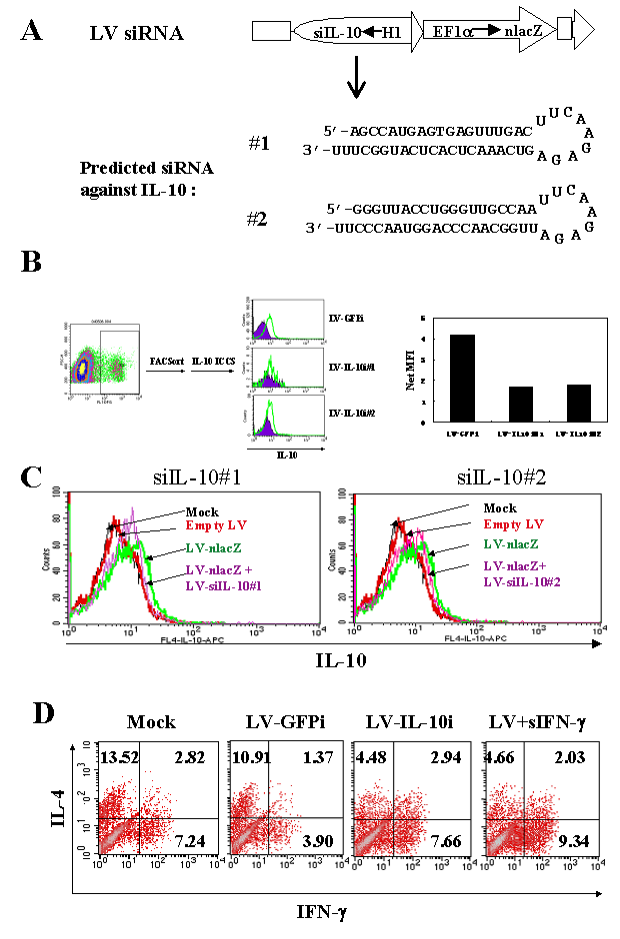
**Modification of DCs by LV-siRNA targeting IL-10. **(A) LV-siRNA targeting IL-10. LV siRNAs targeting two different sites of IL-10 mRNA were illustrated. The predicted hairpin siRNA structure is shown. (B) Illustration of efficient down-regulation of IL-10 in B lymphocytes after LV IL-10 siRNA transduction. Epstein-Barr virus (EBV) transformed B cells were transduced with LV siRNA targeting IL-10 (#1 and #2) or GFP gene. The siRNA LVs also carry a lacZ reporter gene which could be labeled with fluorescein di-b-D-galactopyranoside (FDG) to separate the transduced from un-transduced cells by FACS sort. (C) Immature DCs were transduced with mock, empty LVs, LV-nlacZ, or LV-nlacZ plus LV-siIL-10 #1 or #2, treated with LPS, and analyzed by ICCS and FACS using anti-IL-10 antibody. (D) Enhanced Th1 response by DCs transduced with LV-siRNA targeting IL-10. DCs were transduced with LVs and either co-transduced with LV-siRNA targeting IL-10 (LV-IL10i#2) or GFP (GFPi) or treated with soluble IFN-γ as controls, and the DCs were then assayed for T-cell activation function by DC:T cell co-culture. The T cells were fully rested before reactivation with PMA and ionomycin after 10 days of co-culture. The numbers shown in the FACS quadrants are percentages of the total gated cell population. Results are representative of three independent experiments.

IL-10 is a critical immune modulatory gene and modulation of IL-10 gene expression may alter DC function. To test this, we constructed LVs encoding siRNA targeting IL-10. We chose two regions in the IL-10 mRNA as the siRNA target sites (Fig. 4A). The siRNA expression was driven by a human H1 polIII promoter that was cloned into LVs as previously reported.[24] The LV-siRNA vector also carries a nlacZ reporter gene convenient for vector titer determination and for the identification of transduced cells. To demonstrate the siRNA effects, we transduced B cells with IL-10-siRNA LVs or a control siRNA LV targeting GFP gene, and after transduction, the B cells were expanded and the lacZ-positive cells were FACS-sorted using fluorescent substrate FDG. The expression of IL-10 was quantified by ICCS and FACS using anti-IL-10 Ab. The result demonstrated IL-10 suppression in the lacZ-positive B cells that were transduced with LVs expressing the two IL-10 specific siRNAs but not the non-specific siRNA targeting GFP gene (Fig. 4B).

The effect of the IL-10 LV siRNAs was then examined in DCs by co-transduction using a reporter LV and the IL-10 LV-siRNAs. The transduced DCs were then treated with LPS and analyzed for IL-10 expression as described above. Again, the empty LV had no effect and LV transduction alone up-regulated IL-10 expression. However, co-transduction with LV-siRNA targeting IL-10 down-regulated IL-10 expression (Fig. 4C); the low level of IL-10 expression in DCs was expected as the DC culture was derived and maintained in GM-CSF and IL-4 supplemented media.

To examine whether co-transduction of DCs with LVs expressing the IL-10 siRNA could promote a Th1 response, we transduced DCs with LV alone or together with either an LV-siRNA (#2) or a control LV-siRNA (GFPi). For positive control, we incubated DCs with soluble IFN-γ as previously described. After the DCs were co-cultured with naïve T cells for 10 days, the T cells were reactivated and analyzed for Th functions by ICCS to determine intracellular expression of IFN-γ and IL-4. The results clearly demonstrated that the IL-10 LV-siRNA vector, but not the GFPi LV-siRNA vector, enhanced Th1 response at levels comparable to that of the positive control (DCs treated with soluble IFN-γ, Fig. 4D).

## Discussion

Although HIV-1 is an immunopathogen in humans, HIV-1 derived vectors do not contain viral genes and have been rendered replication-defective. In this study, we found that LV transduction of DCs resulted in altered DC surface marker phenotypes. These changes in DC phenotypes led to suppressed function in mediating the Th1 immunity. DCs transduced by LVs did not lose the capacity to stimulate allogeneic T-cell proliferation, as reported by others [13,14,16]. However, in the DC:T cell co-culture functional assay, we showed that after LV transduction, DCs had significantly reduced ability to polarize naïve CD4+ T cells to differentiate into Th1 effectors. The changed gene-expression profile of DCs after LV transduction correlates with Th1 suppression. As demonstrated here, DC-mediated immunity requires antigen presentation, T cell co-stimulation, and cytokine production, all of which were down-regulated upon LV infection. These results are consistent with a recent study demonstrating cultured immature DCs and DCs from 6 of 10 HIV-1 patients display reduced maturation function and diminished MLR in DC:T cell coculture [28].

Cytokines have critical roles in shaping up the immune response [31,32]. We have detected up-regulation of IL-10 in HUVEC, B cells and DCs after LV infection suggested possible immune suppression by LVs (data not shown). Earlier work has shown that IL-10 inhibits the expression of IL-12 and co-stimulatory molecules in DCs,[32] a finding that correlates with its ability to inhibit the primary T-cell response and induce a stage of anergy in allo- or peptide-antigen-activated T cells [33]. IL-10 has also been shown to down-regulate ICAM-1 in human melanoma cells [34]. Here we showed that LV transduction of DCs, led to down-regulation of CD80, CD86, and ICAM-1. Many of these immune regulatory genes are activated through transcriptional factor NF-κB. Using cDNA microarray analysis, we detected reduced NF-κB expression in DCs after LV infection (not shown), suggesting that LV infection may trigger a cascade of immune suppression through down-regulation of the NF-κB signaling pathway.

It has been reported that HIV-1 Tat up-regulates IL-10 as a result of intranuclear translocation of NF-κB and activation of the protein kinases ERK1 and ERK2 [35]. However, the LVs used in this study do not carry a tat gene. The fact that empty LV particles did not induce the same effects as did intact LVs, suggests that Tat or other virion-associated proteins do not play a role. Thus, it is plausible that events after retroviral attachment and fusion, such as reverse transcription and integration, might trigger the observed cellular response. It would be interesting to see if such immune suppression also occurs *in vivo *following LV gene transfer.

DCs, during their interaction with T cells, provide multiple signals to polarize naïve T cells. These signals include the co-stimulatory molecules CD80, CD86, and ICAM-1, which are considered "signal 2" for T-cell stimulation. The roles of these co-stimulatory molecules on Th differentiation remain controversial. Many studies have shown that ICAM-1 promotes Th1 commitment [36]. CD80 and CD86 have been reported to polarize CD4^+ ^T cells toward the Th2 subset through engagement with CD28 [37-39]. However, CD80 could also interact with CTLA-4 to induce Th1 polarization [40]. Moreover, CD86 has been reported to be a Th1-driving factor [41]. Further studies are needed to address the roles of co-stimulatory molecules in the development of DC and T-cell immunity. Nevertheless, the down-regulation of T cell co-stimulatory molecules in DCs after LV transduction could potentially have an impact on the DC-mediated Th1 response.

The analysis of surface-marker expression profile also revealed down-regulation of CD1a and DC-SIGN in DCs after LV transduction. CD1a is a nonpolymorphic histocompatibility antigen associated, like MHC class I molecules, with beta-2-microglobulin, and is responsible for the presentation of lipid antigens. DC-SIGN (DC-specific, ICAM-3 grabbing nonintegrin) is a 44-kDa type I membrane protein with an external mannose-binding, C-type lectin domain [42]. It has been postulated that DC-SIGN interacts with ICAM-3 on T cells to allow sufficient DC-T cell adhesion and, in addition, that DC-SIGN is a new member of the co-stimulatory molecule family [5,43]. With these characteristics, the down-regulation of CD1a and DC-SIGN might also contribute to the impaired Th1 function of DCs.

Polarization of naïve Th cells into Th1 cells is critical for the induction of cellular immunity against intracellular pathogens and cancer cells. The observed impairment of the Th1 response by LV-transduced DCs raises a potential issue with LV-based immunotherapy. We illustrated that co-transduction with LV encoding IL-12 or IFN-γ, but not CD80, CD86, or ICAM-1, in DCs effectively restored Th1 immunity. In addition, co-transduction with LVs expressing small interfering RNA targeting IL-10 could also promote DC-mediated Th1 immunity. In a step toward future generation of vaccines, LVs encoding IL-12 and IL-10-siRNA as potent Th1 adjuvants may be used to enhance the cellular immune response in the prime-and-boost vaccination regimen. In summary, our study has addressed an important immune suppression effect of LVs and presented a solution that is important for future LV-based DC immunotherapy applications.

## List of abbreviations used

HIV-1 human immunodeficiency virus type 1

LV lentiviral vector

DC dendritic cell

Th T helper

MLV murine leukemia virus

RT-PCR reverse transcription-polymerase chain reaction

FACS fluorescence activated cell sorter

ICCS intracellular cytokine staining

IL interleukin

PLAP placenta alkaline phosphatase

siRNA small interfering RNA

PBMC peripheral blood mononuclear cells

MOI multiplicity of infection

LPS lipopolysaccharide

TNFα tumor necrosis factor alpha

INF-γ interferon-gamma

## Competing interests

The author(s) declare that they have no competing interests.

## Authors' contributions

The study was conceived by LJC; XC and LJC participated in designing and coordinating the study; JH carried out some of the lentiviral constructions, siRNA designs and participated in result discussion; XC performed the statistical analysis; LJC and XC carried out detailed analysis of the results and XC drafted and LJC finalized the manuscript. All authors read and approved the final manuscript.
